# Not Just a One-Way: Mahaim Accessory Pathway Concomitantly Supporting Orthodromic Atrioventricular Re-Entrant Tachycardia

**DOI:** 10.3390/jcm12010159

**Published:** 2022-12-25

**Authors:** Alexandru Deaconu, Viviana Gondos, Radu Vatasescu

**Affiliations:** 1 Faculty of Medicine, Carol Davila University of Medicine and Pharmacy, 050474 Bucharest, Romania; 2 Cardiology Department, Clinical Emergency Hospital, 014461 Bucharest, Romania; 3 Department of Medical Electronics and Informatics, Polytechnic University of Bucharest, 060042 Bucharest, Romania

**Keywords:** Mahaim accessory pathway, atrioventricular reentry tachycardia, electroanatomical 3D mapping

## Abstract

Introduction: We report the case of a 41-year-old female with documented narrow QRS tachycardia. During electrophysiological study, both orthodromic and antidromic atrioventricular reentry tachycardia (AVRT) were demonstrated as well as short episodes of pre-excited atrial fibrillation. Programmed atrial stimulation resulted in decremental anterograde conduction on the AP, thus confirming an unexpected Mahaim accessory pathway (AP) diagnosis. Discussion: Limited 3D activation maps of the right atrium during orthoAVRT, respectively, and the right ventricle (RV) during antiAVRT were constructed and helped accurately describe the atrial and ventricular insertion points, which were superposed on the tricuspid ring, confirming the existence of a single short atrio-ventricular right free wall AP. Short atrioventricular APs with anterograde Mahaim-type conduction concomitantly sustaining orthodromic AVRT are extremely rare. Conclusions: Electroanatomical 3D mapping may help both to clarify the diagnosis and increase the success rate by accurately describing the insertion points of complex accessory pathways.

## 1. Introduction

Mahaim and Winston first described in 1941 the histology of anomalous connections that arise from the atrioventricular (AV) node and insert into the right ventricle (RV) [1]. Later, it was demonstrated that decrementally conducting connections can be between the right atrium (RA) or the AV node and the RV in or close to the right bundle branch (RBB) [2,3]. Although Mahaim accessory pathways (APs) are anatomically distinct from the nodoventricular pathway, they present similar electrocardiographic and electrophysiological characteristics [4], because of the slow rate of recovery of excitability, and they are adenosine-sensitive [5]. Typically, Mahaim APs sustain antidromic atrioventricular re-entrant tachycardia (AVRT) with a left bundle branch (LBBB) morphology [4]. We will discuss the rare case of a short atrio-ventricular AP with anterograde Mahaim-type conduction which is concomitantly sustaining orthodromic AVRT.

## 2. Case Presentation

**Figure 1:**
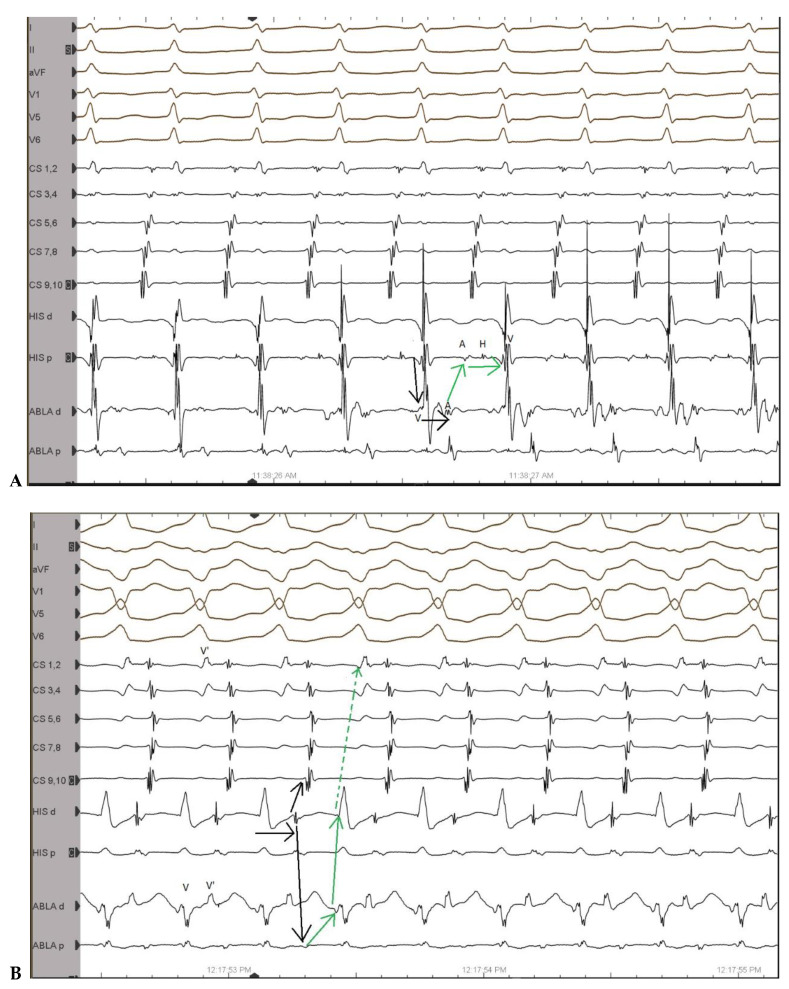
(**A**): Orthodromic AVRT—Shortest VA interval on the ablation catheter without VA fusion; (**B**): antidromic AVRT; ECG leads from the top: DI, DII, aVF, V1, V5, V6. His d and His p indicate distal and proximal His bundle electrogram; CS 1-10, coronary sinus distal to proximal; Abla d and p, ablation catheter distal and proximal, respectively, placed in the RV; A = atrium, H = His, V = ventricle, V’ = far-field ventricle; black and green arrows indicate the sequence of activation.

A 41-year-old female with documented narrow QRS tachycardia which was termed orthodromic AVRT presented for ablation of the AP. During electrophysiological (EP) study, both orthodromic as well as antidromic AVRT were demonstrated (Figure 1A,B).

**Figure 2:**
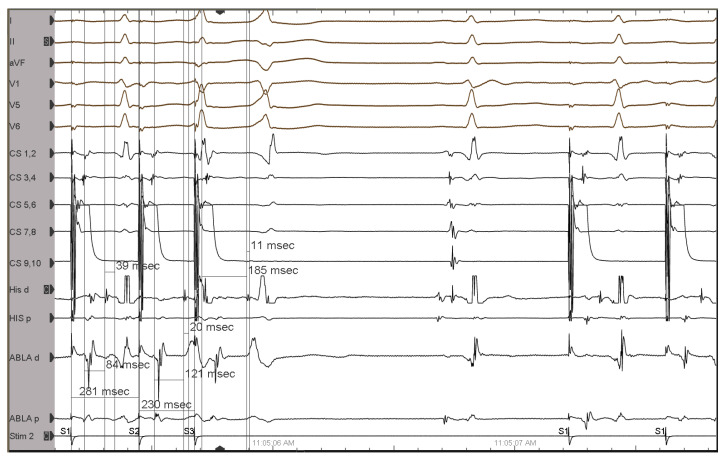
Programmed atrial stimulation with two premature stimuli: An extrastimulus is introduced from the coronary sinus at 340 ms after the last pacing drive stimulus (cycle length of 600 ms). This results in minimal pre-excitation: wider QRS, with a delta wave better visible in DI predominately negative in aVF and V1 (positive during the pacing drive and sinus rhythm); a second extrastimulus is delivered 10 ms earlier than the first (330 ms) and results in an atrial-His bundle (A-H) interval of 125 ms, longer than the A-H interval during the pacing drive (84 ms), and a His-ventricular (H-V) interval of 11 ms, shorter than that observed during the pacing drive (39 ms) de-fining an even greater degree of pre-excitation, with a clearly wider QRS. That electrophysiological behavior is the so-called Mahaim physiology of the accessory pathway. ECG leads from the top: DI, DII, aVF, V1, V5, V6. His d and His p indicate distal and proximal His bundle electrogram; CS 1-10, coronary sinus distal to proximal; Abla d and p, ablation catheter distal and proximal, respectively, placed in the RV.

Programmed atrial stimulation resulted in decremental conduction over the AP. During the programmed atrial stimulation with two premature stimuli, the HV interval shortened while the AH interval increased (Figure 2), thus confirming the Mahaim AP diagnosis.

**Figure 3:**
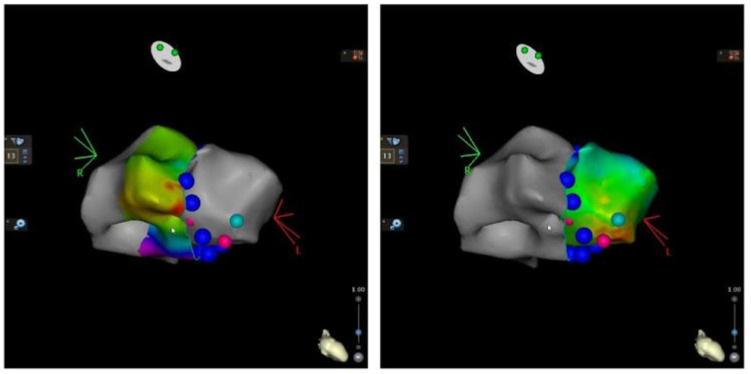
Limited 3D atrial mapping during orthodromic AVRT (**left**) and limited 3D ventricular activation mapping during antidromic AVRT (**right**); pink dots: earliest atrial and ventricular signals, respectively; dark-blue dots: tricuspid annulus; light blue dots: mark the limit from which ventricular electrograms no longer precede the surface QRS.

**Figure 4:**
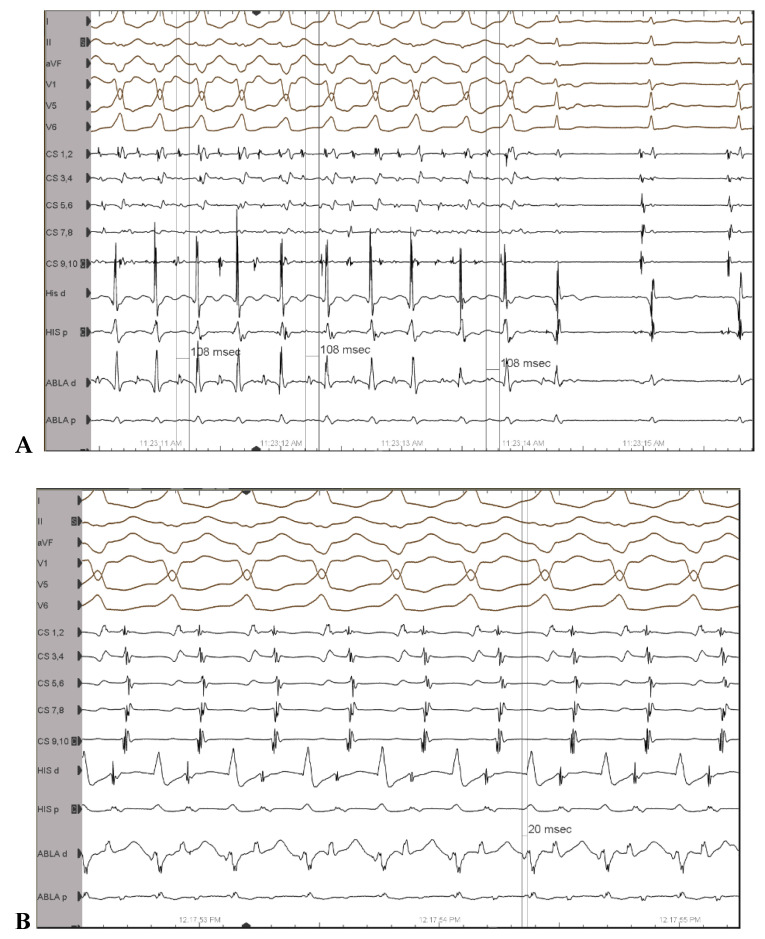
(**A**): Accessory pathway potential recorded on the AV ring during pre-excited atrial fibrillation. (**B**): Accessory pathway potential during antidromic AVRT; ECG leads from the top: DI, DII, aVF, V1, V5, V6. His d and His p indicate distal and proximal His bundle electrogram; CS 1-10, coronary sinus distal to proximal; Abla d and p, ablation catheter distal and proximal, respectively, placed in the RV.

3D activation maps were constructed as follows: an atrial activation map during or-thodromic AVRT and a ventricular activation map during antidromic AVRT in order to accurately describe the insertion points of the Mahaim AP (Figure 3). This resulted in an unusual anatomy of the AP, as it proved to be a short AP, situated at 7 o’clock in LAO projection and close to the tricuspid annulus. AP potentials were also recorded on the ventricular side of the tricuspid ring (Figure 4). 

**Figure 5:**
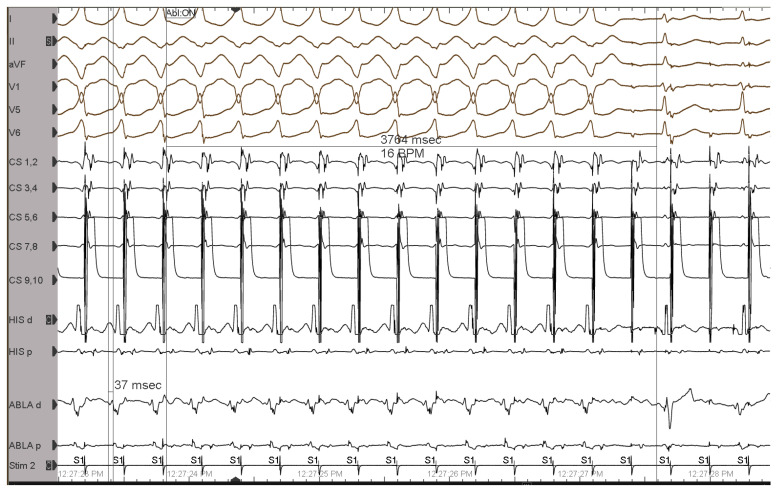
During full pre-excitation elicited by fast atrial pacing (CL 290 ms), radiofrequency application at the ventricular insertion of the accessory pathway induced a loss of pre-excitation in 3.5 s; ECG leads from the top: DI, DII, aVF, V1, V5, V6. His d and His p indicate distal and proximal His bundle electrogram; CS 1-10, coronary sinus distal to proximal; Abla d and p, ablation catheter distal and proximal, respectively, placed in the RV.

Consequently, efficient ablation of the AP was facilitated by a Agilis NxT steerable introducer (St. Jude Medical, St. Paul, MN, USA) and performed successfully by targeting the distal ventricular insertion (Figure 5). An automatic rhythm with similar morphology to that of the clinical tachycardia occurred during radiofrequency delivery at the point of successful ablation. The tachycardia was rendered non-inducible, and there was no evidence of the persistence of the AP.

## 3. Discussion

Mahaim APs represent an atypical form of APs that are rare in clinical practice. The term Mahaim APs is commonly used to report decrementally conducting connections between the right atrium or the atrioventricular (AV) node and the right ventricle in or close to the right bundle branch [4], or its distal Purkinje network [5]. Consequently, pathways with Mahaim characteristics can be atriofascicular, atrioventricular, nodofascicular, or nodoventricular, depending on their variable proximal and distal insertions, and the term Mahaim is widely used as an umbrella term for pathways with characteristic electrophysiological properties, long conduction times, and rate-dependent conduction. During ventricular activation through the Mahaim AP, it results in antidromic re-entrant tachycardia, with a left bundle branch block morphology [5]. The baseline ECG of patients with Mahaim APs generally shows minimal or no pre-excitation (as in our patient) because of conduction through the atrioventricular node, due to the long conduction time over the AP [5].

Mahaim APs are typically decremental and conduct only anterogradely [4]. Rarely, retrogradely conducting nodoventricular pathways has been described [6,7]. Whether such pathways are classified as ‘true’ Mahaims is a matter of terminology rather than essence [4]. Moreover, patients with Mahaim APs often have accompanying APs or dual-node physiology with AV nodal re-entrant tachycardia [8]. In one large study, 9 patients out of 55 patients with Mahaim AP were found to have other types of supraventricular tachyarrhythmias in addition to MAP during the electrophysiological study: 6 patients with AV re-entrant tachycardia, 2 with AV nodal re-entrant tachycardia, and 1 with atrial tachycardia [9]. Additionally, this study found that 43 out of 55 cases were ablated at the free wall tricuspid anulus. In our case, an associated concealed AV pathway which would explain the orthodromic AVRT was excluded by electroanatomical 3D mapping. The mapping of atrial insertion of the AP during orthodromic AVRT and the mapping of the ventricular insertion of the AP during programmed atrial stimulation with pre-excitation were performed and the two insertions correspond over the tricuspid valve ring. To the best of our knowledge, this is the first report in which 3D electroanatomical atrial and ventricular mapping confirmed that a single short right free wall atrioventricular AP with anterograde Mahaim properties can sustain both antidromic as well as orthodromic AVRT. In a complex AP, 3D electroanatomical mapping helps to identify the insertion points of the AP, reduce the fluoroscopy times, and accurately perform ablation at the insertion sites. Additionally, 3D-guided ablation allows the operator to return to the target zone and apply consolidating radiofrequency lesions in cases with catheter instability even after temporary loss of accessory pathway conduction.

In Mahaim APs, catheter ablation is performed by identifying the proximal and distal insertions [4]. Furthermore, recording of a proximal pathway potential at the tricuspid annulus or a distal one on the right ventricular free wall would be ideal [4]. Atrial pacing may facilitate pathway potential recording [4]. In 80% of cases, successful ablation is from the atrial side, while in the remaining cases. Ablation needs to be performed at the ventricular insertion [10]. In our case, we decided to perform ablation at the ventricular insertion since the accessory pathway potential was recorded at this site. Ablation at the ventricular insertion site should be carried out proximal to the pathway’s first connection with the ventricle or right bundle to avoid prolonging the VA conduction time and escalating episodes of AVRT [11].

## 4. Conclusions

In rare cases of complex and/or atypical accessory pathways, electroanatomical 3D mapping should be used both to clarify the diagnosis and increase the success rate by accurately describing the insertion points of the pathway.

## Data Availability

Data available on request due to restrictions eg privacy or ethical The data presented in this study are available on request from the corresponding author. The data are not publicly available due to General Data Protection Regulation.
